# Predictors of acute throat or esophageal patient reported pain during radiation therapy for head and neck cancer

**DOI:** 10.1016/j.ctro.2018.08.004

**Published:** 2018-09-04

**Authors:** Hiram A. Gay, Jung Hun Oh, Aditya P. Apte, Mackenzie D. Daly, Douglas R. Adkins, Jason Rich, Peter J. Oppelt, Pawel T. Dyk, Daniel F. Mullen, Laura Eschen, Re-I. Chin, Brian Nussenbaum, Bruce H. Haughey, Wade L. Thorstad, Joseph O. Deasy

**Affiliations:** a Department of Radiation Oncology, Washington University School of Medicine, St. Louis, MO, United States; b Department of Medical Physics, Memorial Sloan Kettering Cancer Center, New York, NY, United States; c Division of Medical Oncology, Washington University School of Medicine, St. Louis, MO, United States; d Alvin J. Siteman Cancer Center, Washington University School of Medicine, St. Louis, MO, United States; e Department of Otolaryngology, Washington University School of Medicine, St. Louis, MO, United States; f Department of Radiation Oncology, Missouri Baptist Cancer Center, St. Louis, MO, United States; g American Board of Otolaryngology, Houston, TX, United States; h Head and Neck Surgery, Florida Hospital Celebration Health, Celebration, FL, United States

**Keywords:** Head and neck, Pain, Radiotherapy, Esophagus, Throat

## Abstract

**Background and purpose:**

Acute pain during weekly radiotherapy (RT) to the head and neck is not well characterized. We studied dose-volume metrics and clinical variables that are plausibly associated with throat or esophageal pain as measured with a weekly questionnaire during RT.

**Materials and methods:**

We prospectively collected weekly patient-reported outcomes from 122 head and neck cancer patients during RT. The pain score for each question consisted of a four-level scale: none (0), mild (1), moderate (2), and severe (3). Univariate and multivariate ordinal logistic regression analyses were performed to investigate associations between both esophageal and throat pain and clinical as well as dosimetric variables.

**Results:**

In multivariate analysis, age was significantly associated with both types of pain, leading to odds ratio (OR) = 0.95 (p = 0.008) and OR = 0.95 (p = 0.007) for esophageal and throat pain, respectively. For throat pain, sex (OR = 4.12; p = 0.010), with females at higher risk, and fractional organ at risk (OAR) mean dose (OR = 3.30; p = 0.014) were significantly associated with throat pain.

**Conclusions:**

A fractional OAR mean dose of 1.1 Gy seems a reasonable cutoff for separating no or mild pain from moderate to severe esophageal and throat pain. Younger patients who received RT experienced more esophageal and throat pain. Females experienced more throat pain, but not esophageal pain.

## 1 Introduction

Radiotherapy (RT) to head and neck causes mucositis and pain in most patients by the end of the treatment course. Treatment-related pain results from radiation damage to the mucosal epithelium, causing thinning, atrophy, inflammation, and resulting ulceration [1]. The pain can be worsened by radiation-induced xerostomia and reduced mucosal lubrication, and in some cases superimposed candida or bacterial infection [1]. Mucositis seems to involve five biological phases: initiation, primary damage response, signal amplification, ulceration, and healing [2]. Erythema, an early sign of mucositis, presents around 4–5 days following chemotherapy or 10 Gy or more of radiation [3]. Confluent ulcers develop 7–10 days after chemotherapy or after 30 Gy of radiation given in 2 Gy fractions [3], coinciding with an increase in pain. The addition of cytotoxic chemotherapy to RT has been reported to be associated with worse oral mucositis than RT alone [4].

A qualitative study noted that all participants viewed effective pain management as a key facet of their RT treatment for head and neck cancer, previous pain experienced influenced current perceptions of pain, forewarning of potential pain did not reliably improve pain experiences, and participants preferred and benefited from pain management by a specialist team [5].

Tumor, dental extraction, neoadjuvant chemotherapy, or head and neck surgery-related pain may be present prior to the initiation of radiation, thereby complicating the patient’s pain experience during radiation. Chang et al. have found that transdermal fentanyl reduced pain during RT, but with increased nausea and vomiting [6]. In a phase III randomized trial comparing doxepin rinse versus placebo, Leenstra et al. have shown that doxepin diminished oral mucositis pain [7]. In contrast, Ling and Larsson have found that individualized pain treatment with systemic analgesics maximally exploited was insufficient to reduce pain severity [8].

Acute pain during weekly RT is not well characterized or understood, despite the resulting significant impact on quality of life. Given this knowledge gap, we studied dose-volume metrics and clinical variables that are plausibly associated with throat or esophageal pain as measured with a weekly questionnaire during RT.

## 2 Materials and methods

We prospectively collected weekly patient-reported outcomes (PROs) from 122 consecutive head and neck cancer patients during RT who were treated at Washington University School of Medicine in St. Louis between 2010 and 2012. The study was approved by institutional review board. After removing patients who received multiple RT and who did not have target RT structures analyzed in this current study, 96 patients were evaluable. The majority of patients (N = 94) were treated with intensity-modulated RT (IMRT); only two patients were treated with 3D conformal RT (3DCRT). The questionnaire was designed to measure the degree of pain in 16 anatomical structures: gums/gingiva, lip, lymph node(s), ear, eye, face, food pipe/esophagus, mouth, neck, scalp, sinus, skin, throat, tongue, tooth/teeth, and voice box/larynx. Patients were asked: “Do you have pain in the:”. The pain score for each question consisted of a four-level scale: none (0), mild (1), moderate (2), and severe (3). The current study focused on 2 of the 16 anatomical locations: the “food pipe/esophagus” and the “throat”. The organ at risk (OAR) contouring was standardized. The esophagus was contoured inferiorly from the level of the sternal notch to its superior extent. The throat was contoured inferiorly from the inferior border of the mandible superiorly to the hard palate and encompassed the oral cavity.

Patients were treated in the supine position while immobilized using a thermoplastic mask. Fusion of PET/CT and/or MRI scans to the planning CT helped define the clinical tumor volumes (CTVs) as well as clinical and pathologic information. Up to two CTVs (CTV1, CTV2) were defined. In general, for surgical patients, the CTV1 encompassed the high-risk volume which consisted of the pre-operative primary gross tumor volume (GTV) with a 0.5–1 cm margin and any involved lymph node levels. For non-surgical patients, the CTV1 encompassed the primary GTV with a 1–1.5 cm margin and involved lymph nodes plus a 0.5 cm margin. For both types of patients, the CTV2 corresponded to electively treated lymph node levels. Planning target volumes (PTVs) were defined by adding 0.5 cm to the corresponding CTVs and subtracting 3 mm from the skin. Depending on the treatment, CTV1 and CTV2 received 70 and 56 Gy (non-surgical), 66 and 54 Gy (surgical p16-), or 60 and 52 Gy (surgical p16+), respectively. Patients receiving chemotherapy received: either induction or concurrent chemotherapy. Concurrent chemotherapy consisted of either cisplatin, carboplatin, or cetuximab. Induction chemotherapy included TPF (docetaxel, cisplatin, and 5-FU), ACCF (Abraxane, Cetuximab, Cisplatin, and 5 –FU), or carboplatin and etoposide. In general, pain medications were prescribed as needed starting with “magic mouthwash” (aluminum hydroxide and magnesium hydroxide, diphenhydramine elixir, viscous lidocaine, and nystatin in equal parts swish and swallow), followed by an opioid prescribed on an as-needed basis, and finally, a combination of a fentanyl patch for baseline pain and oxycodone or morphine as needed for breakthrough pain.

### 2.1 Statistical analysis

Univariate and multivariate ordinal logistic regression analyses were performed to investigate associations between both esophagus and throat pain and clinical as well as dosimetric variables. Dosimetry data was extracted from the esophagus and oral cavity planning volumes using CERR (computational environment for radiological research) [9]. Because peak pain levels are typically reached well before the end of treatment, we tested “fractional” OAR dose-volume metrics obtained by dividing dose-volume histogram metrics by the number of fractions. The endpoint was the maximum pain score derived from the weekly PROs.

## 3 Results

Weekly completion rates of PROs were 79%, 82%, 83%, 80%, 81%, 79%, and 64% for esophageal pain and 81%, 84%, 88%, 82%, 80%, 77%, and 70% for throat pain. Table 1 shows patient characteristics. Regarding sex, out of 96 patients, 75 were male and 21 were female. At the time of RT consultation, 21 were smokers. There were 26 heavy drinkers and 57 patients with ≥20 pack-year smoking history. Forty-seven patients received chemotherapy and 47 patients underwent surgery. Most patients (N = 63) received RT to both sides of the neck and 20 patients were treated on one side of the neck whereas 13 patients received no neck RT; for those patients, only the primary PTV was irradiated without intentional neck radiation. Forty-six patients required a feeding tube at any time and 31 of those patients still had one at the last follow-up. The most common primary tumor sites were oropharynx and larynx with 33 and 20 patients, respectively. The T stage of most patients (N = 57) was T3 or T4. The N stage of most patients (N = 52) was N2.

**Table 1:** Patient characteristics. Numbers in parentheses indicate standard deviation.

Variable	Category	Esophagus pain			Throat pain		
		Maximum pain score: 0 or 1 (*n* = 49)	Maximum pain score: 2 or 3 (*n* = 47)	p	Maximum pain score: 0 or 1 (*n* = 38)	Maximum pain score: 2 or 3 (*n* = 58)	p
Mean age (years)		61.7 (11.0)	58.4 (11.2)	0.260	63.1 (10.1)	58.1 (11.5)	**0.020**

Sex (*n*)	Male	40	35	0.396	33	42	0.094
	Female	9	12		5	16	

Median CTV (Gy)		66	70	0.066	66	70	0.068

Side of neck treated (*n*)	None	10	3	0.054	10	3	**0.001**
	Either side	12	8		11	9	
	Both sides	27	36		17	46	

G-tube required at any time (*n*)	No	34	16	**0.001**	29	21	**<0.001**
	Yes	15	31		9	37	

G-tube permanent at last follow-up (*n*)	No	37	28	0.095	31	34	**0.019**
	Yes	12	19		7	24	

Alcohol (*n*)	Rarely or never	24	11	**0.023**	21	14	**0.007**
	Occasional	16	19		11	24	
	Heavy (2 or more drinks per day)	9	17		6	20	

Smoking (*n*)	Never	17	8	0.055	12	13	0.261
	<20 pack years	4	10		3	11	
	≥20 pack years	28	29		23	34	

Chemotherapy (*n*)	No	31	18	**0.014**	26	23	**0.006**
	Yes	18	29		12	35	

Surgery (*n*)	No	17	32	**0.001**	13	36	**0.008**
	Yes	32	15		25	22	

Neck dissection (*n*)	None	25	36	**0.029**	21	40	0.274
	Either side	15	8		10	13	
	Both sides	9	3		7	5	

Smoker at consult (*n*)	No	39	36	0.723	30	45	0.874
	Yes	10	11		8	13	

Fractional mean dose (Gy)		0.73 (0.54)	0.99 (0.41)	**0.006**	1.02 (0.53)	1.58 (0.42)	**<0.001**

Site (*n*)	Hypopharynx	1	2	0.100	1	2	**0.001**
	Larynx	11	9		9	11	
	Nasal Cavity	6	2		7	1	
	Nasopharynx	3	3		2	4	
	Oral Cavity	6	5		2	9	
	Oropharynx	10	23		6	27	
	Salivary Gland	5	2		5	2	
	Skin	3	0		3	0	
	Unknown	4	1		3	2	

T-stage (*n*)	T1	8	5	0.275	6	7	0.511
	T2	8	11		5	14	
	T3	9	12		10	11	
	T4	18	18		13	23	
	Unknown	6	1		4	3	

N-stage (*n*)	N0	15	9	0.162	14	10	0.145
	N1	2	6		2	6	
	N2	26	26		16	36	
	N3	2	5		3	4	
	Unknown	4	1		3	2	

CTV: clinical tumor volume. For p-value calculation, Wilcoxon rank-sum test and Chi-square test were used for continuous and categorical (or ordered) variables, respectively. Bold numbers indicate a statistically significant P value.

**Fig. 1:**
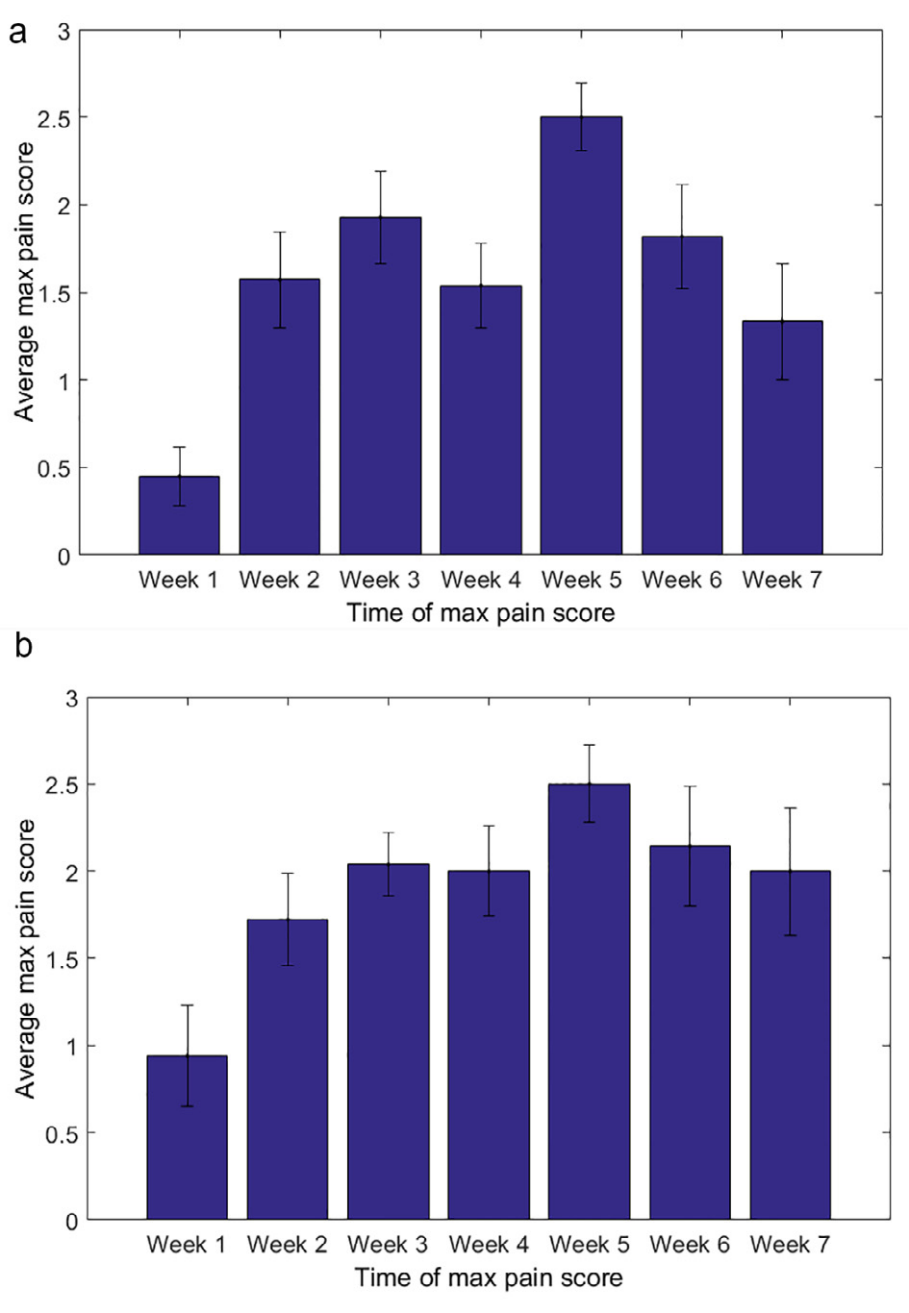
Average maximum pain score at each week after the start of radiotherapy for (A) esophagus pain and (B) throat pain. The error bar indicates the standard error.

**Fig. 2:**
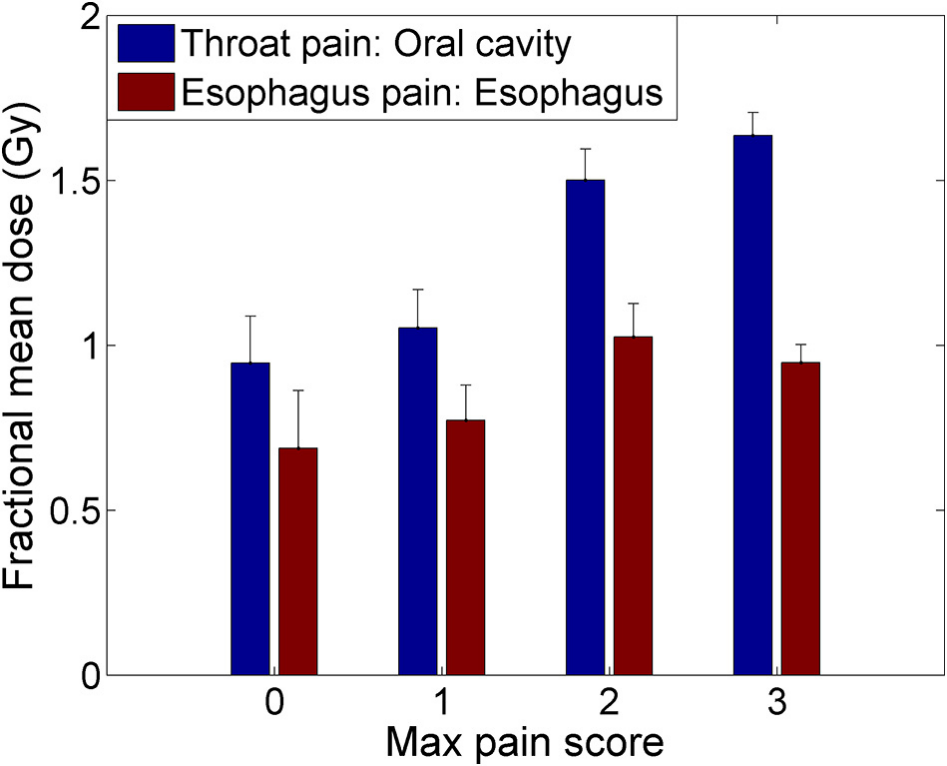
Fractional mean dose in the esophagus for esophagus pain and in the oral cavity for throat pain as a function of maximum pain scores. The error bar indicates the standard error.

**Fig. 3:**
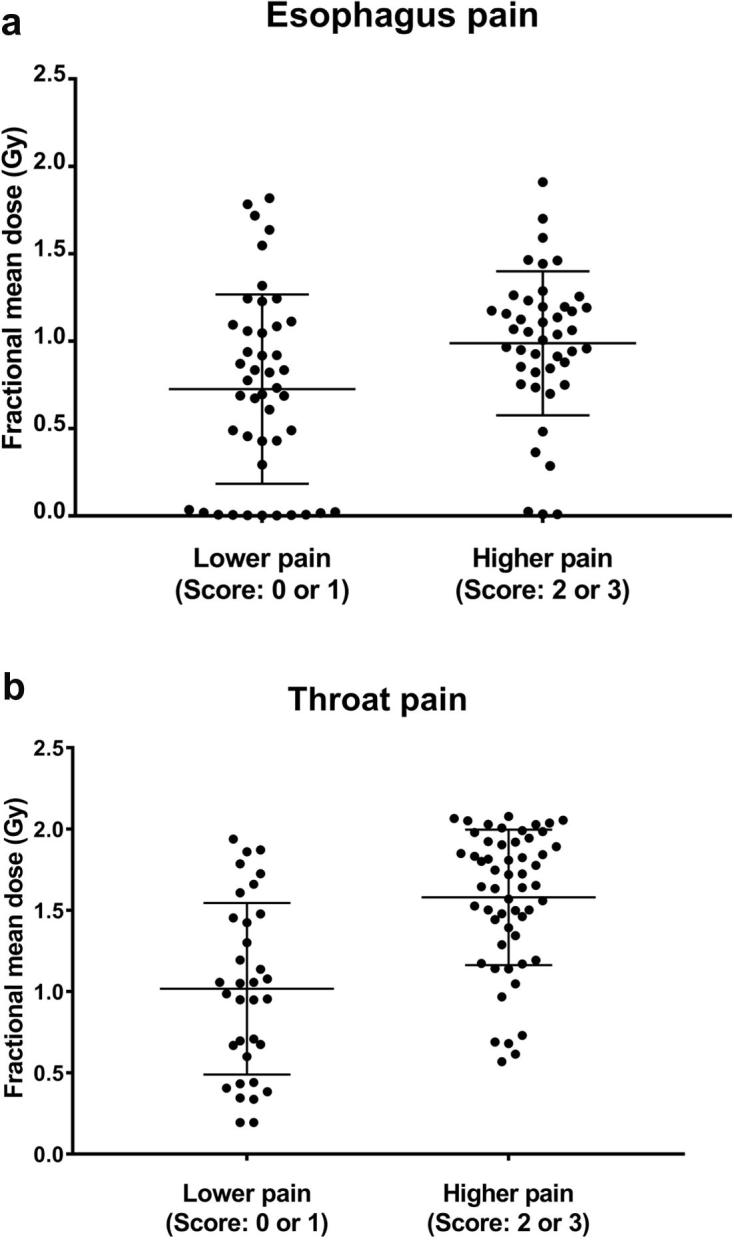
Patients were dichotomized into lower pain (score: 0 or 1) and higher pain (score: 2 or 3) groups. Logistic regression analysis resulted in odds ratio = 3.1 (95% confidence interval [CI]: 1.2–7.9; p = 0.015) and odds ratio = 10.2 (95% CI: 3.6–29.2; p < 0.001) for esophagus pain and throat pain, respectively. This means that for each increase in 1 Gy of fractional organ at risk mean dose, the estimated odds of experiencing higher pain increase by a factor of 3.1 and 10.2, respectively.

For this cohort, maximum pain scores were averaged for each treatment week as shown in Fig. 1. For both types of pain, the pain score reached its peak, on average, in the 5th week, with an average pain score of 2.5 (standard error (SE): 0.19) for esophageal pain and 2.5 (SE: 0.22) for throat pain, respectively. Fig. 2 shows the fractional mean dose in the esophagus for esophageal pain and the oral cavity for throat pain as a function of maximum pain scores. Overall, a trend was observed where pain scores increase as fractional OAR mean doses increase with Spearman correlation coefficients of 0.26 (p = 0.014) and 0.50 (p < 0.001) for esophageal and throat pain, respectively. Using Fisher’s exact test, the best cutoff in fractional OAR mean dose that separates those patients who had an esophageal pain score of 0 or 1 from those with 2 or 3 was 1.09 Gy (p = 0.001) whereas it was 1.06 Gy (p < 0.001) for throat pain (Fig. 3). For simplicity, we summarize this using 1.1 Gy as the fractional OAR mean dose cutoff for both pain endpoints.

In univariate ordinal logistic regression using Dx (minimum dose to the x% highest dose volume), mean dose, and maximum dose in esophagus, mean dose showed the highest odds ratio (OR) associated with esophagus pain: OR = 2.40 (p = 0.027) (see Table 2). For throat pain, maximum dose in the oral cavity showed the highest OR of 19.55 (p = 0.006) followed by mean dose with an OR of 6.64 (p < 0.001). In univariate analysis, significant clinical variables were found to be associated with both types of pain, including age, side of neck treated, alcohol, chemotherapy, and surgery (see Table 3). Sex was significantly associated with throat pain (OR = 2.58; p = 0.043) and neck dissection with esophageal pain (OR = 0.54; p = 0.021) which shows that patients with neck dissection in either side had less pain than those with no surgery and patients with neck dissection in both sides had less pain than those with neck dissection in one side. Chemotherapy did not seem to impact pain on multivariate analysis. Due to the heterogeneity of induction and concurrent chemotherapy regimens used, it is difficult to reach a meaningful analysis on the impact of a specific chemotherapeutic agent or strategy.

**Table 2:** Univariate ordinal logistic regression for Dx (minimum dose to the x% highest dose volume), mean dose, and maximum dose in the esophagus and oral cavity for esophagus pain and throat pain, respectively. The variables are fractional dose-volume metrics.

	Esophagus pain		Throat pain	
Variable	Odds ratio	p	Odds ratio	p
D5	2.04	**0.016**	6.43	**0.002**
D10	1.98	**0.023**	5.70	**0.001**
D15	1.90	**0.035**	5.61	**<0.001**
D20	1.90	**0.039**	5.43	**<0.001**
D25	1.94	**0.037**	5.28	**<0.001**
D30	1.99	**0.034**	5.13	**<0.001**
D35	2.04	**0.032**	4.96	**<0.001**
D40	2.09	**0.028**	4.84	**<0.001**
D45	2.13	**0.026**	4.80	**<0.001**
D50	2.17	**0.027**	4.76	**<0.001**
D55	2.24	**0.024**	4.75	**<0.001**
D60	2.29	**0.023**	4.72	**<0.001**
D65	2.32	**0.026**	4.71	**<0.001**
D70	2.28	**0.038**	4.70	**<0.001**
D75	2.23	0.061	4.62	**<0.001**
D80	2.22	0.089	4.52	**<0.001**
D85	2.15	0.140	4.56	**<0.001**
D90	2.11	0.196	4.85	**<0.001**
D95	1.99	0.296	4.92	**<0.001**
D100	2.20	0.299	5.76	**0.002**
Mean dose	2.40	**0.027**	6.64	**<0.001**
Maximum dose	1.91	**0.022**	19.55	**0.006**

Bold numbers indicate a statistically significant P value.

**Table 3:** Univariate and multivariate ordinal logistic regression analyses using clinical variables and fractional mean dose.

	Esophagus pain				Throat pain			
Variable	Univariate		Multivariate		Univariate		Multivariate	
	OR	p	OR	p	OR	p	OR	p
Age	0.96	**0.030**	0.95	**0.008**	0.96	**0.033**	0.95	**0.007**
Sex	1.49	0.367			2.58	**0.043**	4.12	**0.010**
CTV	1.00	0.695			1.00	0.158		
Fraction in CTV	1.01	0.526			0.99	0.577		
Side of neck treated	1.77	**0.031**	1.05	0.917	3.18	**<0.001**	1.84	0.148
Alcohol	1.73	**0.021**	1.46	0.152	1.69	**0.028**	1.32	0.302
Smoking	1.35	0.163			1.14	0.527		
Chemotherapy	2.88	**0.005**	1.54	0.424	3.75	**0.001**	0.85	0.770
Surgery	0.34	**0.005**	1.01	0.986	0.28	**0.001**	0.34	0.061
Neck dissection	0.54	**0.021**	0.48	0.117	0.62	0.065		
Smoker at consult	0.81	0.635			1.06	0.903		
Fractional mean dose	2.40	**0.027**	2.24	0.134	6.64	**<0.001**	3.30	**0.014**

OR: odds ratio; CTV: clinical tumor volume. The variables of ‘side of neck treated’ and ‘neck dissection’ were coded as 0 = none, 1 = right or left, and 2 = both. For sex, male = 0 and female = 1. Bold numbers indicate a statistically significant P value.

Multivariate ordinal logistic regression analysis was performed using clinical variables with p < 0.05 in the univariate analysis and fractional OAR mean dose. Age was significantly associated with both types of pain, leading to OR = 0.95 (p = 0.008) and OR = 0.95 (p = 0.007) for esophagus and throat pain, respectively. This implies that, other factors being equal, younger people tend to experience more pain during RT. For throat pain, sex (OR = 4.12; p = 0.010), with females at higher risk, and fractional OAR mean dose (OR = 3.30; p = 0.014) were significantly associated with throat pain. In addition, ordinal logistic regression analyses were performed on dosimetric and clinical variables for surgical and non-surgical groups, separately. The results are shown in the Supplementary data.

**Fig. 4:**
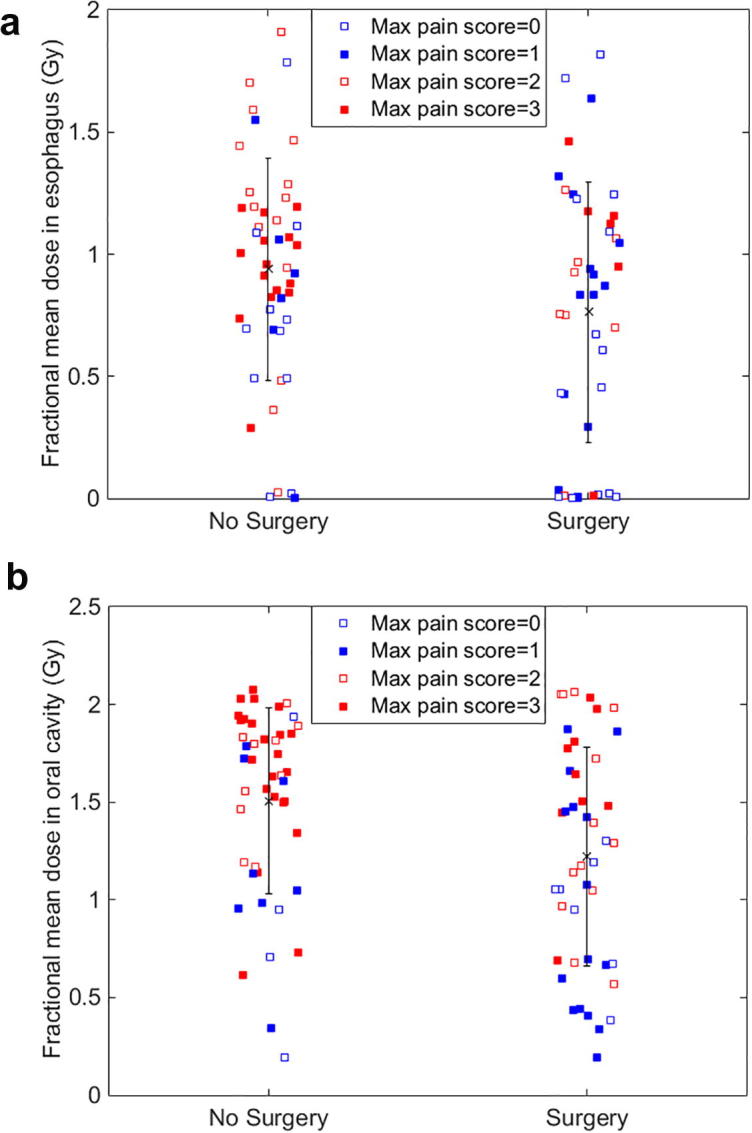
Comparison of fractional mean dose (A) in esophagus for esophagus pain and (B) in oral cavity for throat pain between surgical and non-surgical groups. The error bar indicates the standard deviation.

It was observed that surgical patients had more unilateral neck radiation than non-surgical patients with more bilateral neck radiation (Chi-square test; p < 0.001). There was a statistically significant difference of fractional mean dose in the oral cavity with p = 0.015 using the Wilcoxon rank-sum test between the surgical (lower fractional OAR mean dose) and non-surgical groups whereas fractional mean dose of the esophagus was not statistically different between the two groups (p = 0.159) (Fig. 4).

## 4 Discussion

To our knowledge, this is the first published evidence of dosimetric predictors associated with pain during head and neck RT, using a substantial cohort of 96 patients. Based on our analyses, we propose that a fractional OAR mean dose of 1.1 Gy is a reasonable cutoff that separates esophageal and throat pain scores of 0–1 from those of 2–3 (see Fig. 3). It is important to note that for each increase in 1 Gy of fractional OAR mean dose, the estimated odds of experiencing higher esophagus or throat pain increase by a factor of 3.1 and 10.2, respectively. Reducing the maximum throat dose, thus reducing mucosal hot spots, may also help reduce throat pain as shown in the highest odds ratio (see Table 2).

The data raise several questions. First, should the mucosal surface be an IMRT optimization structure? A clinically practical way of doing this could entail the following steps: autocontouring the air inside the head and neck (oral cavity, nasal cavity, nasopharynx, oropharynx, and hypopharynx), expanding this volume by 3–4 mm, and subtracting the initial autocontoured air volume. An IMRT goal where the fractional dose to this volume is less than 1.1 Gy could be specified. Depending on the PTV, this fractional OAR mean dose goal may not be feasible. However, knowing the fractional OAR mean dose may be useful for counseling the patient about the level of pain that he or she may experience, and for alerting the physician that more aggressive pain management may be necessary.

Secondly, should elective node prescriptions be reconsidered (CTV2)? As previously described, the CTV1 fractional dose for the surgical and non-surgical scenarios was 2 Gy. However, the fractional dose for the elective CTV2 was 1.6 (56 Gy in 35 fractions) Gy, 1.63 (54 Gy in 33) Gy, and 1.73 (52 Gy in 30) Gy for the non-surgical, surgical p16−, and surgical p16+ scenarios, respectively. Although the total dose decreases in each scenario (which is desirable), the fractional OAR dose actually increases, which, as shown, tends to result in increased acute pain. One could hypothesize that further reducing the fractional dose to consistently deliver 1.6 Gy or less to the CTV2 without compromising clinical outcomes would be desirable (e.g., 52.8 Gy in 33 or 48 Gy in 30 fractions).

This is the first study to our knowledge that shows that younger patients receiving RT experience more esophageal and throat pain, but the reason for this is unclear. We believe that perceptual, pharmacological response, or biological differences influenced by aging could be responsible. This is in contrast to the current evidence from a study by El Tumi et al. that suggests old adults may be more sensitive to mechanically evoked pain [10]. Females experienced more throat pain, but not esophageal pain. This discrepancy is not clearly explained either. However, cumulative evidence suggests that females may experience more severe clinical pain and have, on average, greater pain sensitivity [11].

There was a statistically significant difference of fractional mean dose in the oral cavity between the non-surgical and surgical patients as a result of treatment policies. Surgical patients had a lower fractional OAR mean dose. This is likely because surgical patients had more unilateral neck radiation than non-surgical patients, resulting in a smaller volume of the mucosa receiving radiation. Although the univariate analysis suggested that patients receiving unilateral radiation experienced less pain, the multivariate analysis did not.

Pain increased from week 1 to week 3 during RT (see Fig. 1), reaching a population peak in week 5, and then decreased in weeks 6 and 7 to about week-2 levels. It is not clear why there is a pain decrement in the last two weeks, although it is likely that mucosal epithelium repopulation plays a major role. Other possible factors include increased use of narcotic pain medications, decreased oral intake, the initiation of tube feedings reducing mucosal mechanical trauma, or improved nutrition from feeding tube use resulting in better healing. Our institution has adopted a reactive feeding tube policy: if center-dependent nutritional maintenance requirements are not being fulfilled, the patient undergoes feeding tube placement.

This study has some important limitations. In particular, the first questionnaire was given after up to 4 treatment fractions. However, most patients received the questionnaire before the third fraction. From clinical experience and the lateness of the pain peak, it is unlikely that this would result in any significant increase in the baseline pain score. Also, the patient population includes both surgical and non-surgical patients, with surgical patients receiving two different fractionation schemes. Although anatomical regions were as specific as possible, the descriptor ‘maximum pain’ fails to address the extent of the painful tissue volume. For example, although we suspect that treating the oral cavity unilaterally versus bilaterally may result in a similar maximum pain experience for a patient, the mucosal surface experiencing pain is probably reduced in the former with a corresponding reduction in overall pain experience. Another limitation is that some patients may have difficulty in distinguishing between throat and esophageal pain.

Finally, patients did not keep pain medication diaries, which may have helped to better understand the effect of pain medications. The pain management strategy was fairly homogeneous among patients because they were managed by the same physician, which should minimize this as a confounding factor.

## 5 Conclusions

A fractional OAR mean dose of 1.1 Gy seems a reasonable cutoff for separating no or mild pain from moderate to severe esophageal or throat pain. Younger patients receiving RT experienced more esophageal and throat pain. Females experienced more throat pain, but not esophageal pain. Further studies, including mucosal dose as an IMRT optimization goal, may help reduce the pain experienced by patients, resulting in improved quality of life during treatment.

## 6 Conflict of interest

None to disclose.
