# Supplementary material for: Predictors of acute throat or esophageal patient reported pain during radiation therapy for head and neck cancer
Source: Clin Transl Radiat Oncol. 2018 Sep 4;13:1–6. doi: 10.1016/j.ctro.2018.08.004 (PMC6134163; doi:10.1016/j.ctro.2018.08.004)
Supplement: Supplementary Data 1 [file mmc1.docx]

Supplementary Table 1. Univariate ordinal logistic regression in non-surgical group.

|  | Esophagus pain | | Throat pain | |
| --- | --- | --- | --- | --- |
| Variable | Odds ratio | p | Odds ratio | p |
| D5 | 2.11 | 0.112 | 21.97 | **0.016** |
| D10 | 1.82 | 0.206 | 15.23 | **0.011** |
| D15 | 1.57 | 0.333 | 11.48 | **0.008** |
| D20 | 1.47 | 0.412 | 10.70 | **0.005** |
| D25 | 1.47 | 0.421 | 10.67 | **0.002** |
| D30 | 1.48 | 0.415 | 9.54 | **0.002** |
| D35 | 1.52 | 0.390 | 8.18 | **0.001** |
| D40 | 1.58 | 0.348 | 6.79 | **0.001** |
| D45 | 1.64 | 0.316 | 6.01 | **0.001** |
| D50 | 1.68 | 0.300 | 5.41 | **0.001** |
| D55 | 1.76 | 0.262 | 5.13 | **0.001** |
| D60 | 1.82 | 0.239 | 4.91 | **0.001** |
| D65 | 1.86 | 0.237 | 4.76 | **0.001** |
| D70 | 1.89 | 0.253 | 4.57 | **0.002** |
| D75 | 2.02 | 0.247 | 4.42 | **0.003** |
| D80 | 2.22 | 0.238 | 4.22 | **0.004** |
| D85 | 2.47 | 0.231 | 4.14 | **0.006** |
| D90 | 2.70 | 0.235 | 4.14 | **0.010** |
| D95 | 2.62 | 0.316 | 4.12 | **0.018** |
| D100 | 3.01 | 0.323 | 3.89 | 0.080 |
| Mean dose | 2.02 | 0.239 | 8.72 | **0.001** |
| Maximum dose | 2.20 | 0.098 | 474.32 | **0.017** |

Supplementary Table 2. Ordinal logistic regression for clinical variables in non-surgical group.

|  | Esophagus pain | | | |  | Throat pain | | | |
| --- | --- | --- | --- | --- | --- | --- | --- | --- | --- |
| Variable | Univariate | | Multivariate | |  | Univariate | | Multivariate | |
|  | Odds ratio | p | Odds ratio | p |  | Odds ratio | p | Odds ratio | p |
| Age | 0.94 | **0.009** | 0.97 | 0.262 |  | 0.95 | **0.034** | 0.97 | 0.321 |
| Sex | 1.40 | 0.633 |  |  |  | 3.46 | 0.149 |  |  |
| CTV | 1.00 | 0.950 |  |  |  | 1.00 | 0.218 |  |  |
| Fraction in CTV | 1.00 | 0.785 |  |  |  | 0.98 | 0.303 |  |  |
| Side of neck treated | 1.75 | 0.224 |  |  |  | 3.70 | **0.009** | 1.15 | 0.764 |
| Alcohol | 2.14 | **0.023** | 1.05 | 0.903 |  | 1.36 | 0.352 |  |  |
| Smoking | 1.81 | 0.103 |  |  |  | 0.90 | 0.765 |  |  |
| Chemotherapy | 2.17 | 0.217 |  |  |  | 2.20 | 0.213 |  |  |
| Smoker at consult | 1.13 | 0.832 |  |  |  | 1.73 | 0.353 |  |  |
| Fractional mean dose | 2.02 | 0.239 |  |  |  | 8.72 | **0.001** | 3.76 | **0.035** |

Supplementary Table 3. Univariate ordinal logistic regression in surgical group.

|  | Esophagus pain | | Throat pain | |
| --- | --- | --- | --- | --- |
| Variable | Odds ratio | p | Odds ratio | p |
| D5 | 1.63 | 0.208 | 3.32 | 0.084 |
| D10 | 1.72 | 0.180 | 3.23 | 0.053 |
| D15 | 1.79 | 0.164 | 3.53 | **0.023** |
| D20 | 1.87 | 0.145 | 3.60 | **0.011** |
| D25 | 1.94 | 0.132 | 3.49 | **0.009** |
| D30 | 2.02 | 0.122 | 3.40 | **0.008** |
| D35 | 2.04 | 0.125 | 3.35 | **0.008** |
| D40 | 2.06 | 0.125 | 3.43 | **0.007** |
| D45 | 2.05 | 0.135 | 3.53 | **0.005** |
| D50 | 2.05 | 0.147 | 3.62 | **0.004** |
| D55 | 2.08 | 0.154 | 3.69 | **0.004** |
| D60 | 2.07 | 0.168 | 3.71 | **0.004** |
| D65 | 2.04 | 0.196 | 3.76 | **0.005** |
| D70 | 1.83 | 0.292 | 3.85 | **0.005** |
| D75 | 1.54 | 0.479 | 3.88 | **0.006** |
| D80 | 1.38 | 0.630 | 3.93 | **0.006** |
| D85 | 1.18 | 0.819 | 4.12 | **0.007** |
| D90 | 1.05 | 0.956 | 4.50 | **0.009** |
| D95 | 1.00 | 0.998 | 4.56 | **0.014** |
| D100 | 0.99 | 0.994 | 6.84 | **0.014** |
| Mean dose | 1.97 | 0.210 | 4.55 | **0.006** |
| Maximum dose | 1.51 | 0.249 | 7.01 | 0.103 |

Supplementary Table 4. Ordinal logistic regression for clinical variables in surgical group.

|  | Esophagus pain | | | |  | Throat pain | | | |
| --- | --- | --- | --- | --- | --- | --- | --- | --- | --- |
| Variable | Univariate | | Multivariate | |  | Univariate | | Multivariate | |
|  | Odds ratio | p | Odds ratio | p |  | Odds ratio | p | Odds ratio | p |
| Age | 0.97 | 0.278 |  |  |  | 0.95 | 0.061 |  |  |
| Sex | 2.66 | 0.104 |  |  |  | 4.11 | **0.023** | 4.10 | 0.146 |
| CTV | 1.00 | 0.348 |  |  |  | 1.00 | 0.073 |  |  |
| Fraction in CTV | 1.06 | 0.483 |  |  |  | 1.05 | 0.535 |  |  |
| Side of neck treated | 1.18 | 0.644 |  |  |  | 2.07 | **0.047** | 3.16 | 0.062 |
| Alcohol | 1.06 | 0.871 |  |  |  | 1.85 | 0.093 |  |  |
| Smoking | 0.97 | 0.919 |  |  |  | 1.08 | 0.783 |  |  |
| Chemotherapy | 1.69 | 0.433 |  |  |  | 2.68 | 0.149 |  |  |
| Smoker at consult | 0.81 | 0.775 |  |  |  | 1.03 | 0.965 |  |  |
| Fractional mean dose | 1.97 | 0.210 |  |  |  | 4.55 | **0.006** | 5.13 | **0.033** |
